# Evaluation of Performance and Uncertainty of Infrared Tympanic Thermometers

**DOI:** 10.3390/s100403073

**Published:** 2010-03-31

**Authors:** Wenbin Chung, Chiachung Chen

**Affiliations:** Department of Bio-industrial Mechatronics Engineering, National ChungHsing University, 250 Kuokuang Rd., Taichung 40227, Taiwan; E-Mail: bse@dragon.nchu.edu.tw

**Keywords:** infrared tympanic thermometer, uncertainty, calibration equation

## Abstract

Infrared tympanic thermometers (ITTs) are easy to use and have a quick response time. They are widely used for temperature measurement of the human body. The accuracy and uncertainty of measurement is the importance performance indicator for these meters. The performance of two infrared tympanic thermometers, Braun THT-3020 and OMRON MC-510, were evaluated in this study. The cell of a temperature calibrator was modified to serve as the standard temperature of the blackbody. The errors of measurement for the two meters were reduced by the calibration equation. The predictive values could meet the requirements of the ASTM standard. The sources of uncertainty include the standard deviations of replication at fixed temperature or the predicted values of calibration equation, reference standard values and resolution. The uncertainty analysis shows that the uncertainty of calibration equation is the main source for combined uncertainty. Ambient temperature did not have the significant effects on the measured performance. The calibration equations could improve the accuracy of ITTs. However, these equations did not improve the uncertainty of ITTs.

## 1. Introduction

Body temperature is an indication to express the health condition or pathological state. Measurement of body temperature has become an essentially diagnostic method for medical treatment. There are two traditional methods to measure the body temperature. The first type is the glass mercury thermometer. This thermometer is inexpensive and easy to use. However, the response time is from 3 to 5 minutes. The glass material is extremely fragile and can be dangerous to the human body. The second type is the electronic digital thermometer. Its sensing element is made of a thermistor or resistance detector. This meter can measure the temperature within several seconds. However, the electronic device is affected by aging problems. The sensing elements of digital thermometer still need to have contact with the human body. Several problems exist in the clinical operation. The patient reaction, such as children or infant, could affect the measurement of these contact thermometers.

The best method is to measure the core body temperature, such as the temperature of coronary arteries. However, this is impossible except by using invasive surgical procedures. Recently, many literatures reported that the core temperature can be measured by detecting the positions near the membrane of the ear canal [1]. The infrared tympanic thermometer was developed to serve as a detector for medical applications. The construction and operating principle of infrared tympanic thermometer have been introduced in detail [2,3].

The reliability and accuracy of infrared tympanic thermometer have been discussed by many researchers. Their results are inconsistent. Dodd *et al.* [4] compared the reading values of infrared ear temperature for children aged between 0 and 18 with that of rectal thermometry. Their conclusions indicated that the infrared ear thermometer would fail to detect fever in 75% of febrile children. Craig *et al.* [5] found that the pooled mean temperature difference for rectal temperature minus infrared ear temperature was 0.3 °C. The significant difference (significance was taken as p < 0.05) was found between two sets of data. These authors suggested that the infrared ear thermometer did not indicate the sufficient agreement with the body temperature measured by rectal temperature. Kistemaker *et al.* [6] evaluated the performance of an infrared forehead thermometer. They concluded that this Sensor Touch meter could work well in stable conditions. The average difference between the infrared forehead thermometer and a rectal sensor ranged from 0.3 to 0.5 °C. Kocoglu *et al.* [7] compared three body temperatures. The rectal and auxiliary temperatures were measured with glass mercury thermometers. The aural temperature was measured by an infrared thermometer. They concluded that the infrared tympanic thermometer could be applied in an emergency room setting. Rosenthal and Leslie [8] compared the accuracy of an electronic and an infrared thermometer with traditional glass mercury thermometry in the 95% confidence level. They found the average difference between the reading values of infrared thermometer and glass thermometer was within 0.1 °C. Stavem *et al.* [9] assessed the accuracy of infrared ear thermometry, by measuring the rectal and esophageal temperature with thermistor thermometer and ear temperature by infrared thermometer. The mean of two ear temperatures had better agreement with the rectal temperatures.

The inconsistency of the measurement may be explained by the performance, confidence level, and uncertainty of the infrared thermometer. The factors affecting the performance of infrared tympanic thermometer were discussed by Heusch *et al.* [10]. Their results indicated that the handedness, sex and age were the significant factors conflicted the accuracy of the ear temperature measurement. Pusnik and Drnovsek [11] found that several factors could affect the performance of infrared ear thermometers; such as the position of thermometer related to the aperture of the blackbody, the drift of a thermometer due to heating, the amount of times probe covers were used and the differences of probe covers.

The calibration of the infrared thermometer is very important to ensure its performance. Because the emissivity of canal is very close to unity, the temperature of a black body cavity is usually served as the standard temperature for calibration. Cascetta [2] developed a blackbody cavity that consisted of a copper cylinder. This cylinder contacted with the copper plate and maintained a constant temperature by circulating water provided by a hot water bath, Pusnik *et al.* [12] compared the measurements of infrared thermometer performed at several blackbodies. Three cavity shapes were found that could be served as suitable standards for IR calibrations. Simpson *et al.* [13] described a commercial ear thermometer calibrator that could be traceable to ITS-90.

Pusnik *et al.* [3] defined some important terms for the measurement of the infrared thermometer. The performance of accuracy is closeness of the measurement result of thermometer and the true value of a measurement. The difference between the average value of several measurements and the true value was called systematic error. The diversity of individual measurements is presented as random errors. However, the variability of measurements was not the only source that induced from the difference between one measured values and average values of several measurements. Other components could produce a variability source for measurement. The uncertainty of measurements have been defined and explained by the guide to the expression of uncertainty [14]. The components of uncertainty of an infrared ear thermometer were listed [3,12]. These components included the repeatability of an IR thermometer, the reference thermometer, the blackbody radiator, the transducer of instrument and the resolution of the IR thermometer.

Recently, uncertainty evaluation had been widely applied for physical and chemical sensors [15–17]. The effects of calibration equation on the measurement performance have been studied [18,19]. It is very useful to study the influence of the factors affecting the performance of the IR thermometer. As far as the authors know, there have been no reports of uncertainty evaluations of an infrared tympanic thermometer.

The objectives of this study are to evaluate the accuracy and to calculate the uncertainty of two types of infrared tympanic thermometer according to ISO GUM (Guide to the expression of uncertainty in measurement). An adequate calibration equation is first established. Then the effect of the calibration equation on the accuracy and uncertainty was compared.

## 2. Equipment and Methods

### 2.1. Infrared Tympanic Thermometer

Two types of infrared tympanic thermometers were adopted in this study. One is the OMRON MC-510 Gentle Temp model (OMRON Co., Japan). The other is the BRAUN IRT-3020 Themoscan model (Braun Co., Germany). The operating procedures for ear temperature measurement were described in detail in their manual. The specifications of the two thermometers are listed in Table 1.

### 2.2. Standard Temperature

The standard temperature was prepared by a temperature calibrator and a blackbody cavity. This standard temperature was simulated as the tympanic temperature to evaluate the performance of two types of infrared thermometers. The standard temperature served as the standard for performance testing.

The model of temperature calibrator is TC-2000 Scan Sense (Instrutek Co., Norway). The operating temperature was ranged from −40 °C to 150 °C. The inner temperature of oil bath for calibration was detected by a four-wire Pt-100 thermometer. The uncertainty of this calibrator is 0.03 °C according to its specifications.

An aluminum cylinder was inserted into the oil bath of the calibrator. The length of the cylinder was 14.5 cm and the diameter was 0.485 cm. The emissivity of this cylinder was assembled to be unity calculated from the calculation equation of blackbody cavity [20].

### 2.3. Testing Procedures

The target temperature for calibration of infrared thermometer was maintained at 34.5, 36.0, 37.5, 39.0 and 40.5 °C. The performance test experiment was executed in an environmental chamber at three levels of ambient temperature: 25 °C, 30 °C and 35 °C. The variation of the setting temperature was controlled within ±1 °C.

Each infrared thermometer was randomly measured at five sets of standard temperatures at the same ambient temperature. Five replicates were made for each standard temperature. As one measurement was made, the infrared thermometer was taken out of the blackbody cavity for five minutes and then put into the cavity again for further measurement.

### 2.4. Data Analysis

The performance of infrared thermometers was assessed by their accuracy and repeatability. The definition of accuracy is the closeness with which a measurement approaches true value [11]. The error is defined as:

(1) Ei=Tr−Ts

where *Tr* is reading value of the infrared thermometer in °C; and *Ts* is standard value of the infrared thermometer in °C.

The standard deviation of data sets was calculated from replicates. These values were then further analyzed by one and two way ANOVAs (Analysis of Variance) and other statistical procedure. Significance was taken as P < 0.05. The P value is the smallest level of significance that would be to reject the hypothesis.

### 2.5. Establish the Calibration

The regression analysis technique was applied to establish the calibration equation from the relationship between the standard values and the reading values. The criteria for selecting of the best equation are the coefficient of determination R^2^, t-tests of each parameters and residual plots [21,22].

To establish the calibration equation, the standard values of the standard temperature made by a temperature calibrator were selected as the dependent value y. The reading values of the infrared thermometer were viewed as an independent variable. The ambient temperature of the experiment testing is assumed as the other independent variable. This calibration equation f(x) is called as the inverse equation [23–25]:

(2) $$y=f\left(x_{i}\right)$$

The form of y(x) can be expressed as follows:

(3) $$y=b_{0}+b_{1}X_{1}+b_{2}X_{1}^{2}$$

where X_1_ is the reading values of infrared thermometer, b_0_, b_1_, b_2_ are parameters.

Many texts dealing with the calibration equation reported that the reading values of the thermometer were viewed as the independent variable and the standard values of temperature were viewed as the dependent variable. This equation is called as the classical equation. However, the inverse equation has shown the better predictive ability [23–25].

## 3. Sources of the Uncertainty for Infrared Tympanic Thermometer

According to the ISO GUM [14], the uncertainty of measurement is evaluated by “Type A” and “Type B” methods. The Type A evaluation of standard uncertainty is the evaluation method by statistical procedure. The Type B evaluation of standard uncertainty is the evaluation method by other information of the measurement and instrument. There are several items of uncertainty source for infrared tympanic thermometer.

### 3.1. The Standard Deviation

The standard uncertainty due to the estimate values of standard deviation of replicates u_x_ is a Type A uncertainty. It was easy to calculate from the replicates of the measurement made using the IR thermometer at different conditions.

### 3.2. The Calibration Equation

The standard uncertainty due to the calibration of infrared thermometer is a Type A uncertainty. The uncertainty in a predicted value u_y_ is calculated by follows:

(4) $$u_{y}=s\sqrt{\frac{1}{p}+\frac{1}{n}+\frac{{\left(y-\bar{y}\right)}^{2}}{\sum \left(y_{i}^{2}\right)-{\left(\sum y_{i}\right)}^{2}/n}}$$

where s is standard deviations of calibration equation, p is the number of replicates in the calibration; and n is the number of the sample data.

### 3.3. Uncertainty of the Reference Temperature

The uncertainty source of reference standard was provided by the manufacturer’s specifications. The distribution of uncertainty was assumed as normal distribution. The estimate of uncertainty for the standard temperature is:

(5) $$u_{\mathrm{ref}}=\frac{U_{\mathrm{ref}}}{\sqrt{2}}$$

where U_ref_ is the uncertainty source of TC-2000 temperature calibrator, and u_ref_ is the uncertainty due to the reference temperature.

### 3.4. Uncertainty Due to Nonlinearity and Repeatability

The source U_non_ due to nonlinearity and repeatability is specified by manufacturers. The variation response for this error source is assumed as a rectangular distribution. The uncertainty due to nonlinear and repeatability u_non_ is calculated as:

(6) $$u_{\mathrm{non}}=\pm \frac{U_{\mathrm{non}}}{2\sqrt{3}}$$

where U_non_ is the uncertainty source due to the nonlinear and repeatability that represents a specification interval provided by the manufacturer; and u_non_ is the uncertainty due to the nonlinear and repeatability made using the IR thermometer.

### 3.5. Uncertainty Due to Resolution

The uncertainty measurement due to resolution is assumed to be a rectangular distribution. It is considered as ±½ of the display scale value. The uncertainty value due to resolution u_res_ is estimated as the follows:

(7) $$u_{\mathrm{res}}=\pm \frac{U_{\mathrm{res}}}{2\sqrt{3}}$$

where U_res_ is the uncertainty source due to the resolution effect, and u_res_ is the uncertainty due to the resolution effect.

### 3.6. Uncertainty Due to Ambient Temperature Variation

The uncertainty source of ambient temperature was not mentioned by manufacturers. In this study, the effect of the ambient temperature on the variation of measurements made using the IR thermometer was evaluated by two-way ANOVA test. If the ambient temperature has significant effects on the variation of measurement, the uncertainty due to the ambient temperature can then be further estimated. The variation response from the temperature variation is assumed a rectangular distribution. The uncertainty due to temperature variation is:

(8) $$u_{\mathrm{tem}}=\pm \frac{U_{\mathrm{tem}}}{\sqrt{3}}$$

where U_tem_ is the uncertainty source due to the ambient temperature; and u_tem_ is the uncertainty due to the ambient temperature.

The uncertainty due to the reference temperature, nonlinearity and repeatability, resolution, and ambient temperature are classified as Type B uncertainty.

These uncertainty effects did not have significant correlations, and the combined standard uncertainty u_c_ then be calculated as follows:

- Uncertainty estimation of a fixed measuring point.
  (9) $$u_{c_{1}}=\sqrt{u_{x}^{2}+u_{\mathrm{ref}}^{2}+u_{\mathrm{non}}^{2}+u_{\mathrm{res}}^{2}+u_{\mathrm{tem}}^{2}}$$
- Uncertainty estimation include the effect of calibration
  (10) $$u_{c_{2}}=\sqrt{u_{y}^{2}+u_{\mathrm{ref}}^{2}+u_{\mathrm{non}}^{2}+u_{\mathrm{res}}^{2}+u_{\mathrm{tem}}^{2}}$$

## 4. Results and Discussions

### 4.1. Performance of OMRON MC-510 Thermometer

**Figure 1.**
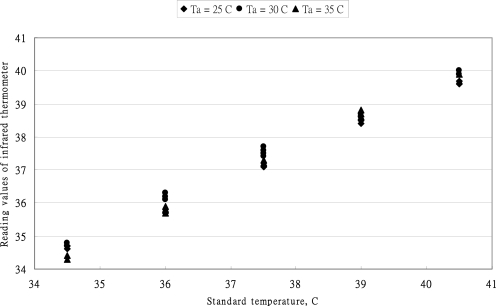
The relationship between reading values of MC-510 thermometer *versus* standard values.

**Figure 2.**
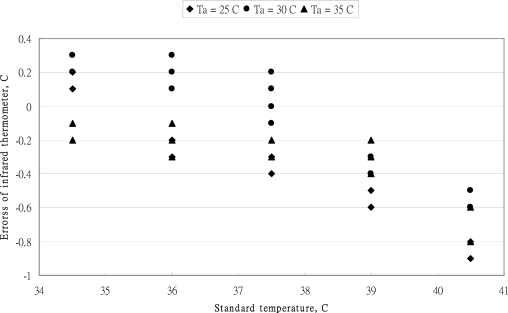
The error distribution of OMRON MC-510 thermometer at three ambient temperatures.

The relationship between reading values of MC-510 thermometer *versus* standard values is shown in Figure 1. The reading values of this tympanic thermometer are close to each other. The effect of ambient temperature on the reading values was inconstant. The error distribution of OMRON MC-510 thermometer at three ambient temperatures is shown in Figure 2. The error band is ±0.3 °C and the error values moves from positive to negative with the increase of standard temperature. According to the standard of the ASTM standard for allowable errors of clinical thermometers is 0.1 °C in the temperature range 37–39 °C, 0.2 °C in the temperature range 36–37 °C and 39–41 °C. The allowable error of clinical thermometers was within a band of ±0.3 °C in the range below 36 °C. From the data distribution of Figure 2, the performance of reading values was corresponding with the requirement of the ASTM standard in the temperature range below 37 °C. However, the reading values were diversity in the temperature of 37.5 °C. Only the average values of several measurements could reduce the measured errors. As the higher temperature ranged higher than 38 °C, the reading values were underestimated.

The relationship between standard temperature values and reading values of MC-510 thermometer was established by regression analysis. The best equation is:

(11) $$\begin{array}{c} T_{\mathrm{sta}}=25.1634-0.4739\;T_{\mathrm{red}}+2.1563\;T_{\mathrm{rea}}^{2}\\ R^{2}=0.992,\;\;\;s=0.1965 \end{array}$$

where T_sta_ is the standard temperature and T_rea_ is the reading temperature of IR thermometer.

**Figure 3.**
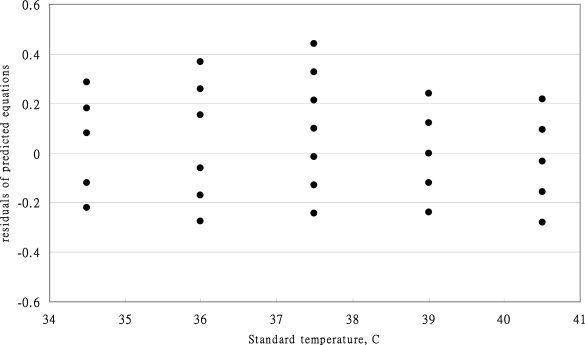
The predicted errors of calibration equation of OMRON MC-510 thermometer.

The predicted errors of calibration Equation (11) are presented in Figure 3. All the predicted errors in the range of standard temperature were below 37 °C and higher than 39 °C all within the ASTM standard. However, the predicted errors at the standard temperature of 37.5 °C revealed high spreading of the measurement. Above the standard temperature of 37 °C, the accuracy of the MC-510 infrared tympanic thermometer had improved significantly.

### 4.2. Performance of BRAUN IRT-3020 Thermometer

**Figure 4.**
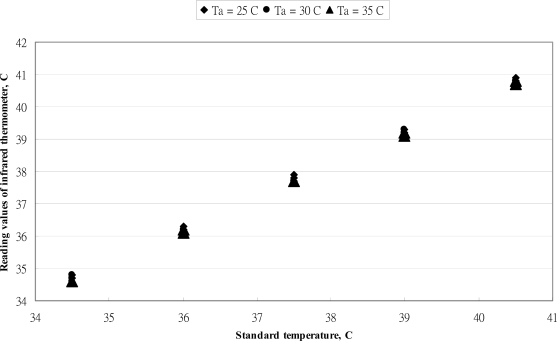
The relationship between reading values of BRAUN IRT-3020 thermometer *versus* standard values.

**Figure 5.**
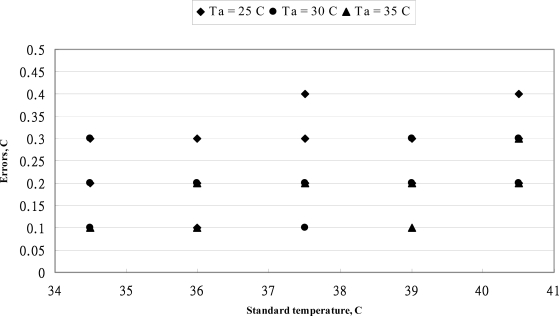
The error distribution of BRAUN IRT-3020 thermometer at three ambient temperatures.

The relationship between reading values of BRAUN IRT-3020 thermometer *versus* standard values is shown in Figure 4. The closeness of the data distribution at each standard temperature indicated a good replication of the performance. The error distribution of this infrared thermometer at three ambient temperatures is presented in Figure 5. The error bands were within 0.2 °C of the whole measuring range except at 37 °C. The reading values of IRT-3020 thermometer could meet the requirements of the ASTM standard in the range below 37 °C. Above the standard temperature of 37 °C, the errors values of this thermometer were higher than the ASTM standard.

The relationship between standard values and reading values of IRT-3020 thermometer was established by regression analysis. The adequate equation is:

(12) $$\begin{array}{c} T_{\mathrm{sta}}=0.2637+0.9871\;T_{\mathrm{red}}\\ \;\;\;R^{2}=0.999,\;\;\;s=0.0696 \end{array}$$

**Figure 6.**
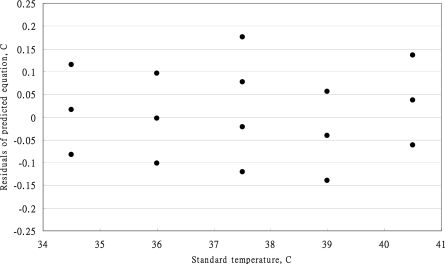
The predicted errors of calibration equation of BRAUN IRT-3020 thermometer.

The predicted errors of Equation (12) are shown in Figure 6. All the predicted errors were located within the requirement range of the ASTM standards except for the standard temperature of 37.5 °C. However, the mean value of several replicates at this temperature could reduce the predictive errors.

### 4.3. The Replicate of the Infrared Tympanic Thermometer

**Figure 7.**
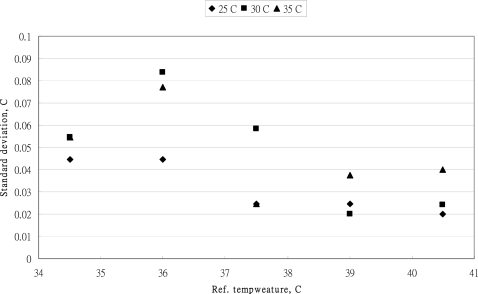
The standard deviation of five measurements of OMRON MC-510 thermometer at different standard temperatures and three leaves of ambient temperatures.

The standard deviation of five measurements of MC-510 thermometer at different standard temperatures and three levels of ambient temperatures are shown in Figure 7.

At the standard temperature of 36 °C, the standard deviations for three ambient temperatures were higher than that of other standard temperatures. To evaluate the factors affecting the standard deviations of MC-510 thermometer, two-way ANOVA table was established and the significant tests were executed by F test with a 95% confidence level. The result is listed in Table 2.

**Figure 8.**
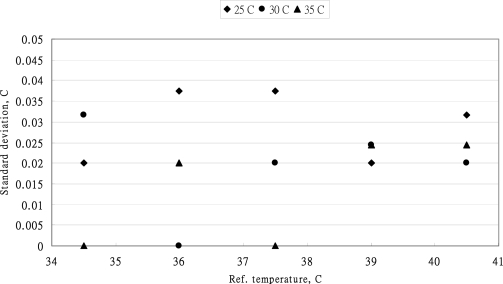
The standard deviation of five measurements of BRAUN IRT-3020 thermometer at different standard temperatures and three leaves of ambient temperatures.

In comparison with the variations of measurements, the standard temperature has significantly affected the variations of standard deviations. However, the ambient temperature showed no significant effects on the variation of measurements (*P* = 0.1305>0.05). The ambient temperature did not influence the variation of standard deviation of each measurement. The uncertainty source of ambient temperature was not considered for the evaluation of uncertainty of the MC-510 thermometer. The distribution of standard deviation of the Braun IRT-3020 measurement is presented in Figure 8. The ambient temperature did not have the significant effect on the standard deviations. At the standard temperature of 39 °C, the standard deviations were closed at 39 °C.

The two-way ANOVA table for the evaluation of the factors affecting the standard deviations of the IRT-3020 thermometer is listed in Table 3. The results indicated that the standard temperatures (*P* = 0.9355 > 0.05) and the ambient temperatures (*P* = 0.2274 > 0.05) did not have the significant effects on the standard deviation of IRT-3020 measurements. The effect of ambient temperature on the uncertainty of IRT-3020 was not considered in further analysis.

### 4.4. Calculation of the Uncertainty of Infrared Tympanic Thermometer

The adequate calibration equation for OMRON MC-510 thermometer is the polynomial equation and the best calibration equation for BRAUN IRT-3020 is a linear form. The uncertainty due to the replications of measurement is the standard deviation of each measurement. However, the uncertainty derived from the calibration equation is calculated by Equation (4). The type A uncertainty of several observations for two-types of infrared tympanic thermometers are listed in Table 4. For the OMRON MC-510 thermometer, the uncertainty of calibration equation did not reduce as compared to the uncertainty calculated from standard deviations of each observation. The same results could be found for the BRAUN IRT-3020 thermometers.

The type B uncertainty analysis for the two infrared tympanic thermometers was calculated by Equation (5), (6) and (7). This result is listed in Tables 5–6. Comparing these sources of uncertainty for two-type infrared tympanic thermometers, the main source of the uncertainty included the specification of nonlinear and repeatability (type B) and the standard deviations (type A).

### 4.5. The Combined Standard Uncertainty

The u_c_ values for two infrared tympanic thermometers at five observations are listed in Table 7.

According to Equation (9), the values of u_c1_ are calculated at 34.5, 36.0, 37.5, 39.0 and 40.5 °C of the standard temperature for MC-510 thermometer. The numeric values are 0.2519, 0.2499, 0.2316, 0.1851 and 0.2318 °C, respectively. For the polynomial form of calibration equation, the combining standard uncertainty calculated by Equation (10) to evaluate at these standard temperatures were 0.2685, 0.2324, 0.2321, 0.2246 and 0.2741, respectively. The result indicated that the polynomial calibration did not decrease the uncertainty for the MC-510 thermometer.

The values of u_c_ are calculated at 34.5, 36.0, 37.5, 39.0 and 40.5 °C of the standard temperature for IRT-3020 thermometer. They are found to be 0.1491, 0.0873, 0.0872, 0.089 and 0.1324 by Equation (9), respectively. For the linear calibration equation, the combined standard uncertainty evaluated at these standard temperatures was 0.1395, 0.0965, 0.0965, 0.0967 and 0.1394 by Equation (10), respectively. This result indicated that the calibration equation did not improve the uncertainty of the IRT-3020 thermometer.

This study evaluated the accuracy and uncertainty for two types of infrared tympanic thermometers. If the reading values of both thermometers were recognized as the true values of tympanic temperature, the accuracy of these thermometers could meet the requirement of the ASTM standards in the temperature range below 37 °C. At the standard temperature of 37.5 °C, there was wide variation in measurement in both thermometers. The average value of several measurements could be used to improve the accuracy. That is, more data needs to be taken at this temperature point to ensure the accuracy. At the temperature range above 37.5 °C, the reading values of both types of infrared tympanic thermometer need to be transformed by calibration equation to improve their accuracy.

The errors of measurement could be classified as systematic and random errors. The systematic errors could be expressed as errors and improved by its calibration equation. These procedures have been studied [18,26]. The random errors can only be improved by selecting of sensing elements or increasing sample numbers. The quantitative expression of this random error has been established in the international standards [14].

In the previous study, the uncertainty of measurement for thermometer, hygrometer and rough rice moisture meter could be improved by establishing the adequate calibration Equations [18, 19, 26]. In this study, the measuring errors could be reduced by the calibration equations. That is the systematic errors could be decreased. However, the uncertainty of measurement for two infrared tympanic thermometers could not be improved by the calibration equations. The reason could be explained by the form of predictive uncertainty [Equation (4)]. The estimated values of standard deviations for calibration, Equations (11) and (12), were 0.1965 and 0.0696, respectively. These numeric values are all almost higher than the standard deviation of replicates for different standard temperature and ambient temperature. That is the reason that the predictive uncertainty calculated by Equation (4) is almost higher than that of standard deviations.

Many studies have been reported that compare the performance of the rectal thermometer and infrared tympanic thermometer [4,5,7]. However, the performance of accuracy and uncertainty of the traditional mercury glass thermometer and the infrared tympanic thermometer were not mentioned by these researchers. That may be the reason for the inconsistency of their results.

## 5. Conclusions

The uncertainty evaluation in this study included the Type A uncertainty for standard deviations of replicates or predicative uncertainty of calibration and the Type B uncertainty for reference temperature, nonlinear and repeatability and resolution source. The ambient temperature variation effect was excluded. The uncertainty sources of different operators or different parts of human body were not discussed in this study. The uncertainty analysis has become the basic information for the instrument. No literature or related reports were found that mentioned the evaluation of infrared tympanic thermometers. The method of uncertainty calculation for two types of infrared tympanic thermometers in this study has been developed. This method could be applied for other infrared thermometers.

## Figures and Tables

**Table 1.** Specification of the infrared clinical thermometers.

	**OMRON MC-510**	**BRAUN IRT-3020**
Sensing element	thermopile	thermopile
Measuring range	34–42.2 °C	34–42.2 °C
Resolution	0.1 °C	0.1 °C
Nonlinearity and repeatability	1. 36.0–39 °C, ±0.2 °C 2. (≤36.0, ≥39 °C), ±0.3 °C	1. 37.0–39 °C, ±0.1 °C 2. (≤37 °C, ≥39 °C), ±0.2 °C

**Table 2.** Analysis of variance ANOVA table for the effect of standard temperature and ambient temperature on the measurement of OMRON MC-510 thermometer.

**Variance**	**SS**	**df**	**MS**	**F**	***P*-value**	***F*-critical value**
Standard temp.	0.003717	4	0.000929	5.9318	0.01614	3.8379
Ambient temp	0.000832	2	0.000416	2.6552	0.1305	4.4590
Errors	0.001253	8	0.000157			
Total	0.005802	14				

Note: SS is the sum of square, df is the degree of freedom, MS is the mean square and F-critical value is the smallest level of significance that be to reject the hypothesis of F test.

**Table 3.** Analysis of variance ANOVA table for the effect of standard temperature and ambient temperature on the measurement of BRAUN IRT-3020 thermometer.

**Source**	**SS**	**df**	**MS**	**F**	***P*-value**	***F*-critical value**
Standard temp.	0.000133	4	3.321E-05	0.1926	0.9355	3.8379
Ambient temp.	0.000617	2	0.000309	1.7924	0.2274	4.4590
Errors	0.001377	8	0.000172			
Total	0.002127	14				

**Table 4.** The type A uncertainty of several observations for two infrared tympanic thermometers.

	**Calibration equations**	**y_obs_ of different temperatures**				
		34.5	36.0	37.5	39.0	40.5
OMRON	None	0.1799	0.2193	0.1767	0.1408	0.1506
MC-510	Polynomial equation	0.2026	0.1990	0.1986	0.1989	0.2100
BRAUN	None	0.0884	0.0535	0.0567	0.0594	0.05606
IRT-300	Linear equation	0.0711	0.0704	0.0701	0.0703	0.0709

**Table 5.** The type B uncertainty analysis for OMRON MC-510 thermometer.

**Description**	**Estimate value**	**Standard uncertainty**	**Probability distribution**
Reference (u_ref_)	0.03 °C	0.0153	Normal
Resolution (u_res_)	0.1 °C	0.0289	Rectangular
Nonlinear and repeatability			
U_non1_ 36–39 °C	0.2 °C	0.1155	Rectangular
U_non2_ ≤ 36 °C, ≥39 °C	0.3 °C	0.1732	

**Table 6.** The type B uncertainty analysis for BRAUN IRT-3020 thermometer.

**Description**	**Estimate value**	**Standard uncertainty**	**Probability distribution**
Reference (u_ref_)	0.03 °C	0.0153	Normal
Resolution (u_res_)	0.1 °C	0.0289	Rectangular
Nonlinear and repeatability			Rectangular
U_non1_ 37–39 °C	0.1 °C	0.0577	
U_non2_ ≤ 37 °C, ≥39 °C	0.2 °C	0.1155	

**Table 7.** The combined uncertainty for two IR thermometers.

**IR thermometer**	**Calibration equation**	**y_obs_ of different temperature**				
		34.5	36.0	37.5	39.0	40.5
OMRON	None	0.2	0.2	0.2	0.1	0.23
MC-510	Polynomial equation	519	499	136	851	18
		0.2	0.2	0.2	0.2	0.27
BRAUN	None	685	324	321	246	41
IRT-300	Linear equation					
		0.1	0.0	0.0	0.0	0.13
		491	873	872	890	24
		0.1	0.0	0.0	0.0	0.13
		395	965	965	967	94
